# Why Is *Plasmodium vivax* a Neglected Tropical Disease?

**DOI:** 10.1371/journal.pntd.0001160

**Published:** 2011-06-28

**Authors:** Jane M. Carlton, Barbara J. Sina, John H. Adams

**Affiliations:** 1 Department of Medical Parasitology, New York University School of Medicine, New York, New York, United States of America; 2 Division of International Training and Research, Fogarty International Center, National Institutes of Health, Bethesda, Maryland, United States of America; 3 Department of Global Health, College of Public Health, University of South Florida, Tampa, Florida, United States of America; Lancaster University, United Kingdom

## Summary

*Plasmodium vivax* malaria is a debilitating, sometimes life-threatening, and economically repressive disease of many tropical and temperate countries outside Africa, and yet it is perceived as relatively benign. The estimated cost of the global burden of vivax malaria is US$1,400,000,000–$4,000,000,000 per year [1], and more people worldwide live at risk from *P. vivax* than *P. falciparum* [2]. All age groups suffer *P. vivax* infections and endure repeated, incapacitating febrile attacks, severe anemia, and respiratory distress, with poor outcomes in pregnancy and learning impairment in children also apparent. Similar to other neglected diseases, *P. vivax* affects primarily poor people lacking access to affordable health care, trapping many in a relentless cycle of poverty because of loss of adult productivity and depletion of meager financial reserves [3]–[5]. Global malaria elimination programs are mobilized against *P. falciparum*, most likely because of the greater mortality rates associated with it, and draw resources away from *P. vivax* even though vivax malaria is harder to prevent, diagnose, and treat, and both species are co-endemic. There is a consensus among malaria experts that eliminating *P. vivax* will prove more technically challenging than eliminating *P. falciparum* [6], and that there exist fewer tools and a weaker knowledge base from which to start an effective global elimination program [7].

## Vivax Malaria: Is Biology Destiny?

A vivax malaria control program will require early diagnosis combined with highly efficacious treatment to cure dormant liver and blood-stage parasites. In addition, disruption of mosquito bite transmission is key, preferably through sustainable community-based vector control programs and routine use of drugs that target the mosquito transmission (“gametocyte”) stages found in human blood. However, case detection is inherently more challenging for vivax malaria due to two of its biological properties. First, the parasite's strong preference to infect the minor population of reticulocytes (immature red blood cells) in the bloodstream results in significantly lower parasitemias, necessitating the use of thick blood smears and greater microscopic skills for proper diagnosis. In many endemic regions *P. vivax* is often overlooked in co-infections with *P. falciparum*, and newly available rapid diagnostic tests have sub-optimal sensitivity for *P. vivax* [8], [9]. A second and more significant problem stems from invisible dormant liver stages that can give rise to multiple periodic “relapse infections” up to several years after an infectious mosquito bite. The unpredictable nature of relapse infections, which can vary from as short as three weeks for tropical strains to five years for strains circulating in temperate climates, further complicates elimination programs since gametocytes typically appear at the earliest onset of clinical symptoms, allowing transmission of *P. vivax* before treatment can be initiated [10].

*P. vivax* control may become even more difficult in coming years as there is increasing prevalence of clinically defined chloroquine-resistant *P. vivax*, for which little monitoring is possible without in vitro culture or a genetic marker for resistance [11]. Equally troublesome are reports that the new frontline treatment of artemisin combination therapy has less impact on *P. vivax* than on *P. falciparum* [12]. Although artemisin combination therapy refractoriness may be largely a consequence of relapse infections from dormant liver stages, there is currently only one class of drug (8-aminoquinolines) with known activity against *P. vivax* dormant liver stages. Unfortunately, 8-aminoquinoline use is limited by contraindications for pregnancy, infancy, and G6PD deficiency—a relatively common genetic condition in many malaria endemic countries for which a point-of-care diagnostic test is not available. These limitations for detection, control, and elimination of *P. vivax* infections indicate a critical need to reduce missed or undetected infections through routine use of gametocidal drugs and/or a *P. vivax* transmission-blocking vaccine. Only two *P. vivax* vaccines have reached phase 1 clinical trials (one a transmission-blocking candidate antigen), compared to 23 *P. falciparum* candidate vaccine antigens, of which several are in phase 2 and one in phase 3 trials [13].

*P. vivax* develops in mosquitoes in temperate climates, thus providing a much broader geographic range of transmission than *P. falciparum*, with more focal, seasonal, and epidemic-driven transmission. Current malaria surveillance, control, epidemiology, mapping, and modeling approaches do not accurately address these transmission patterns. Outside Africa, there is great diversity in malaria mosquito vectors, including species that bite humans primarily outside their homes or early in the evening, limiting the impact of indoor residual spraying and insecticide-treated bed nets. Important *P. vivax* vector species have not been colonized for laboratory research. Levels of insecticide resistance are unknown for most non-African mosquito vectors, and specific genetic and/or biochemical markers of resistance are not defined. The large diversity of anophelines in Asia and the Americas may also pose unanticipated challenges for malaria control, such as rapid adaptation to highly populated urban settings (e.g., *Anopheles stephensi* in India [14]), preference for particular *P. vivax* strains (e.g., *A. albimanus* versus *A. pseudopunctopenis* in Mexico [15]), and zoonotic transmission of primate malarias to humans (e.g., *P. knowlesi* transmission in Malaysia [16]).

## Scientific Neglect? Research Funding, Publication, and Policy

Grant support for *P. vivax* research is unstable and meager. Because of the lack of a continuous in vitro culture system, research on *P. vivax* parasites requires access to infected patients or primates, primarily through collaboration. By its nature, such research proceeds more slowly and is more expensive, logistically challenging, and less competitive when pitted against projects using more modern experimental research methods. The major international tropical disease research sponsors devote a relatively minor portion of their annual malaria research budgets to vivax malaria research. The proportion of the US National Institutes of Health (NIH) malaria research budget devoted to *P. vivax* increased from 3.0% to 5.1% from financial year 2005 to financial year 2009. The tenuous nature of NIH research support for *P. vivax* is apparent during the recent five-year period, as only two of 36 grantees were continuously funded. In contrast, 8.6% of the Wellcome Trust malaria research grants in 2009 focused on *P. vivax* (not including the Southeast Asia Major Overseas Programme). The World Health Organization Special Programme for Research & Training in Tropical Diseases supported only two *P. vivax–* focused grants in the last five years, and currently has no active vivax projects. Recently, the NIH funded ten “International Centers of Excellence for Malaria Research,” which may include a significant focus on vivax research [17].

The lack of research support and the significance of contributions by *P. vivax* endemic country scientists are reflected in publications concerning vivax malaria in peer-reviewed scientific journals (Table S1). PubMed retrieval of malaria articles published between January 1960 and January 2010 indicates that only 12% focused on vivax malaria. Approximately 9% of the malaria papers published by first authors from developed countries concerned *P. vivax,* compared to 20% of the papers published by first authors from developing/endemic countries. Developing country scientists authored a total of 51% of all the vivax papers published during this 50-year span. This proportion likely underrepresents the contribution from vivax endemic country scientists because of the lack of inclusion of many national journals in the PubMed archive.

Malaria research and control policy documents are increasingly including vivax malaria, usually as a minor focus. The Disease Control Priorities in Developing Countries project published a chapter, “Conquering Malaria,” in April 2006 [18], which describes the distribution of vivax malaria and its threat to pregnancy but does not consider vivax in the analysis of morbidity risk, economic impact, implementation of interventions, or research priorities. The 2008 NIH/National Institute of Allergy and Infectious Diseases (NIAID) “Research Agenda for Malaria” [19], which recognized that vivax and other non-falciparum species “…exhibit unique features that may require alternative interventions and strategies…” when considering global elimination of malaria, recommended support for specific vivax malaria activities in four out of nine priority objectives. Roll Back Malaria's “Global Malaria Action Plan” [20], also issued in 2008, and “Shrinking the Malaria Map,” issued in 2009 by the Malaria Elimination Group [6], include more extensive consideration of vivax malaria in research recommendations, as well as control strategies directed to research funders and malaria policy makers (the vivax research recommendations of these documents are compared to those of the vivax malaria research community gathered at the 2009 conference “Vivax Malaria Research III: 2009 and Beyond” in Table 1). A recurrent theme in these documents is the lack of data and interventions specific for vivax malaria upon which evidence-based strategies to tackle vivax malaria can be constructed.

**Table 1:** Comparison of the vivax research recommendations of three malaria research and control policy documents.

Description	“Vivax Malaria Research III: 2009 and Beyond” Recommendations	NIH/NIAID “Research Agenda for Malaria”	Roll Back Malaria “Global Malaria Action Plan”
**Year**	2009	2008	2008
**URL**	http://www.vivaxmalaria.com/template_meetings.htm	http://www.niaid.nih.gov/topics/Malaria/Documents/researchagenda.pdf	http://www.rbm.who.int/gmap/
**Enabling technologies**	Robust culture systems	Develop and characterize an in vitro culture system	
	Animal models		
	Standardized methodologies		
	Mathematical models		
	Genomics tools	Functional comparison between malaria species genomes to define genetic determinants of relapse	
			Operational research to determine when and where mass drug administration is appropriate, and which drugs work best and minimize resistance
			Low-cost, consistently accurate RDT
			Quality assurance system for RDTs and microscopy
			New protocols for primaquine and chloroquine use in areas where resistance is common
			Strong behavioral change communication program to ensure adherence to primaquine treatments
**Critical biological knowledge**	Hypnozoite biology	Hypnozoite mechanisms of dormancy and activation	
	Host–vector ecology	Identify mosquito vectors and effective control tools/interventions	
	Host–parasite interactions	Mechanisms of pathogenesis	
	Cross-species interactions		
	Intervention impact analysis		
		Mine genome to identify possible targets for rational drug design	
		Develop a new class of anti-malarial drug with better safety and pharmacodynamic profile while retaining anti-hypnozoite and anti-gametocyte activity	More treatments that target vivax malaria
		Identify new target antigens as vaccine candidates	New vaccine that specifically targets *P. vivax* alone, or in combination with a *P. falciparum* vaccine component; vaccines that block transmission
**Capacity building**	Networks: regional associations		Regional communication or technical networks to share monitoring data and best vivax control practices
	Networks: regional elimination groups		
	Networks: regional conferences		
	Training: vector biology		
	Training: primate models		
	Training: public health		
		Establish facilities to monitor emergence of drug resistance in endemic countries	

Vivax research emphases and recommendations of the 2008 NIH/NIAID “Research Agenda for Malaria” [19] and the Roll Back Malaria “Global Malaria Action Plan” [20] are shown compared to those of the vivax malaria research community gathered at the “Vivax Malaria Research III: 2009 and Beyond” conference in Panama, May 2009. *P. vivax* research and development recommendations are eagerly awaited from the Bill and Melinda Gates Malaria Eradication Research Agenda. RDT, rapid diagnostic test.

## What Can *PLoS Neglected Tropical Diseases* Do?

In the world of scientific publishing, *PLoS Neglected Tropical Diseases (PLoS NTDs)* was designed to serve as a clarion for scientific progress on tropical diseases neglected by the mainstream journals. *P. vivax* clearly meets the journal's definition of a neglected tropical disease as defined in the journal's scope: “[Neglected tropical diseases] are a group of poverty-promoting chronic infectious diseases, which primarily occur in rural areas and poor urban areas of low-income and middle-income countries. They are poverty-promoting because of their impact on child health and development, pregnancy, and worker productivity…” (http://www.plosntds.org/static/scope.action). *P. vivax* manuscripts should be treated the same as all other manuscripts submitted to *PLoS NTDs,* and not allocated to a special category and selected on a case-by-case basis, as is the current editorial policy. This additional level of scrutiny may dissuade endemic country scientists—the very authors that are particularly encouraged to submit their research to *PLoS NTDs*—from submitting it. A scientific publication “home” for reporting vivax research results at *PLoS NTDs*, one easily accessible online, will provide greater cohesion and information exchange among the vivax research community, thinly scattered across the globe, because the *PLoS NTDs* editors are known to make special efforts to encourage publication from endemic country authors. It is also hoped that the high profile of *PLoS NTDs* will foster greater awareness of the need to specifically address vivax malaria—and other non-falciparum species that infect humans—in the quest to eliminate malaria world wide.

## Supporting Information

Table S1 **Analysis of economic status of country of origin of first authors of** ***P. falciparum*** **and** ***P. vivax*** **articles catalogued in PubMed, 1960–2010.**
(DOC)
