# Supplementary material for: Why Is Plasmodium vivax a Neglected Tropical Disease?
Source: PLoS Negl Trop Dis. 2011 Jun 28;5(6):e1160. doi: 10.1371/journal.pntd.0001160 (PMC3125139; doi:10.1371/journal.pntd.0001160)
Supplement: Table S1 — Analysis of economic status of country of origin of first authors of P. falciparum and P. vivax articles catalogued in PubMed, 1960–2010. (DOC) [file pntd.0001160.s001.doc]

**Supplemental Table 1. Analysis of country of origin economic status of first authors of *P. falciparum* and *P. vivax* articles published in PubMed, 1960-2010**.

PubMed was used to retrieve articles with *'Plasmodium falciparum'* (Pf) or *'Plasmodium vivax'* (Pv) in their MeSH terms, excluding articles having both, yielding 12,261 Pf and 2,482 Pv articles. The MEDLINE field 'AD' (first author affiliation) was used to derive country of article origin. Country of origin economic status was derived from the IMF 2009 World Economic Outlook ([www.imf.org/external/pubs/ft/weo/2009/02/weodata/groups.htm](http://www.imf.org/external/pubs/ft/weo/2009/02/weodata/groups.htm)). Only articles with an AD field were included in the final classification of 9,511 Pf and 1,352 Pv articles, shown below.

| **Country of article origin economic status** | **No. Pf** | **No. Pv** | **Total** | **% Pf** | **% Pv** |
| --- | --- | --- | --- | --- | --- |
| Developing/emerging | 2,686 | 666 | 3,352 | **80** | **20** |
| Developed | 6,825 | 686 | 7,511 | **91** | **9** |
| **Total** | 9,511 | 1,352 | 10,863 | **88** | **12** |
| **% Developing/emerging** | **28** | **49** |  |  |  |
| **% Developed** | **72** | **51** |  |  |  |
